# Factors associated to depression and anxiety in medical students: a multicenter study

**DOI:** 10.1186/s12909-016-0791-1

**Published:** 2016-10-26

**Authors:** Fernanda Brenneisen Mayer, Itamar Souza Santos, Paulo S. P. Silveira, Maria Helena Itaqui Lopes, Alicia Regina Navarro Dias de Souza, Eugenio Paes Campos, Benedita Andrade Leal de Abreu, Itágores Hoffman II, Cleidilene Ramos Magalhães, Maria Cristina P. Lima, Raitany Almeida, Mateus Spinardi, Patricia Tempski

**Affiliations:** 1 Center of Development of Medical Education, School of Medicine of Univrsity of São Paulo, Av. Dr. Arnaldo, 455, Sala 2349, 01246-903 São Paulo, SP Brazil; 2 Department of Pathology, School of Medicine of University of São Paulo, Av. Dr. Arnaldo, 455, Sala 2349, 01246-903 São Paulo, SP Brazil; 3 University of Caxias do Sul, Rua Francisco Getúlio Vargas, 1130, 95070-560 Caxias do Sul, Rio Grande do Sul Brazil; 4 Psychiatry and Legal Medicine Department, School of Medicine of the Federal University of Rio de Janeiro, Rua Professor Rodolpho Paulo Rocco 255, 9° andar, sala 9E28, Rio de Janeiro, 21941-590 RJ Brazil; 5 University Center of Serra dos Órgãos, Av. Alberto Torres, 111, 25964-000 Rio de Janeiro, RJ Brazil; 6 Bionuclear Medicine of Teresina, Rua Desembargador Pires de Castro, 489, 64001-390 Teresina, PI Brazil; 7 School of Medicine of Tocatins, Avenida NS 15, 109, sala 18, 77020-210 Palmas, TO Brazil; 8 Department of Education and Humanities, Federal University of Health Sciences of Porto Alegre , Rua Sarmento Leite, 245, sala 412, 90050-170 Porto Alegre, RS Brazil; 9 Neurology and Psychiatry Department, School of Medicine of São Paulo State University, Rua Rubião Júnior, caixa-postal: 540, 18618-000 Botucatu, SP Brazil; 10 School of Medicine of Federal University of Rondônia , BR 364 km 9,5, 78900-000 Porto Velho, RO Brazil; 11 Marília Medical School, Av. Monte Carmelo, 800, sala 17, 17519-030 São Paulo, Brazil; 12 Center of Development of Medical Education, Avenida Dr Arnaldo, 455, 1 andar, sala 1210, 01246903 São Paulo, Brazil

**Keywords:** Medical student, Depression, Anxiety, Tuition scholarship

## Abstract

**Background:**

To evaluate personal and institutional factors related to depression and anxiety prevalence of students from 22 Brazilian medical schools.

**Methods:**

The authors performed a multicenter study (August 2011 to August 2012), examining personal factors (age, sex, housing, tuition scholarship) and institutional factors (year of the medical training, school legal status, location and support service) in association with scores of Beck Depression Inventory (BDI) and State Trait Anxiety Inventory (STAI).

**Results:**

Of 1,650 randomly selected students, 1,350 (81.8 %) completed the study. The depressive symptoms prevalence was 41 % (BDI > 9), state-anxiety 81.7 % and trait-anxiety in 85.6 % (STAI > 33). There was a positive relationship between levels of state (*r* = 0,591, *p* < 0.001) and trait (*r* = 0,718, *p* < 0.001) anxiety and depression scores. All three symptoms were positively associated with female sex and students from medical schools located in capital cities of both sexes. Tuition scholarship students had higher state-anxiety but not trait-anxiety or depression scores. Medical students with higher levels of depression and anxiety symptoms disagree more than their peers with the statements “I have adequate access to psychological support” and “There is a good support system for students who get stressed”.

**Conclusions:**

The factors associated with the increase of medical students’ depression and anxiety symptoms were female sex, school location and tuition scholarship. It is interesting that tuition scholarship students showed state-anxiety, but not depression and trait-anxiety symptoms.

## Background

The global prevalence of depression among medical students was recently estimated to be 28.0 % according to a meta-analysis of 77 studies [1]. A high prevalence of anxiety and depression among medical students has been reported worldwide [2–19]. An increased prevalence compared with age-matched peers in general population [20, 21] and with non-medical students has been reported in the literature [22].

A number of personal and institutional factors may contribute to the worsening of medical students’ mental health. Recent research discussed that medical schools provide a toxic psychological environment [23–25] where academic pressure, workload, financial hardships, sleep deprivation are stressors factors [2, 26]. Depression and anxiety symptoms carry impairment to medical students, including poorer in academic performance, drop out, substance abuse and suicide [14, 15, 26, 27]. Moreover poor mental health is a predictor of later distress in the physician [27, 28].

While there is a growing literature on prevalence of anxiety and depression symptoms and about potential causal factors to the high prevalence of anxiety and depressive symptoms among medical students, few studies have had a large enough sample and focused on prevalence rates related to both depression and anxiety symptoms in a multicenter study design [2, 3, 29].

In our study we aimed (a) to investigate the prevalence of depression and anxiety symptoms among medical students in 22 Brazilian medical schools; (b) to study their association with personal factors (age, sex, housing, tuition scholarship) and institutional factors (year of the medical training, school legal status (public/private), location and support service). This baseline examination is part of the VERAS Project (acronym for Life of students and residents from health professions).

## Methods

### Study design and sample

VERAS study is a multicenter study involving 22 Brazilian medical schools to evaluate quality of life, emotional competencies and educational environment of students and residents of health professions [30–32].

The participating schools were selected by convenience and were geographically distributed across the country, with a diverse legal status and locations (13 public and 9 private schools; 13 in state capital cities and 9 in other cities).

The sample size (*n* = 1,152) was initially calculated to enable an effect size of 0.165 between two groups of the same size, with 80 % power at a 0.05 significance level. Later, we increased the sample to 1,650 students to account for 30 % of loss of participants. At least 60 medical students, stratified in clusters by gender and program year (i.e., 5 males, 5 females per each of the six training years) were randomly selected using a computer-generated list of random numbers [30–32]. The participation in the study was voluntary without any financial compensation. All participants signed an informed consent form in which confidentiality were guaranteed.

### Data collection

Data were collected from August 2011 to August 2012 through a survey platform. The randomly selected students received a link by e-mail to access the questionnaires and a full 10 days were provided to answer the survey. Once all questionnaires were answered each student received an individual and immediate feedback online for his/her scores. The participants had the opportunity to contact coordinator researchers for guidance and/or emotional support [30–32].

### Instruments

Socio-demographic is a 14-item questionnaire to access age, sex, year of medical training, tuition scholarship and housing.

Beck Depression Inventory (BDI) is a 21-item questionnaire to measure depression symptoms. Each item scores vary from 0 to 3 according to increasing symptom intensity [32]. The cut-offs for the BDI scores were defined as: no depression (0 to 9), mild (10 to 17 points), moderate (18 to 29 points) and severe (30 to 63 points) [33, 34]. This questionnaire was translated to Brazilian Portuguese and demonstrates adequate reliability and validity [33]. The BDI had a Cronbach’s alpha of 0.87 in our study.

State Trait Anxiety Inventory (STAI) is a two-component scale with 20 items each evaluating the intensity of state-anxiety and frequency of trait-anxiety [35]. State-anxiety refers to a transitory emotional state which intensity may vary according to the context and over-time. It is characterized by unpleasant feelings of tension or apprehension and increased activity of the sympathetic nervous system as tachycardia, sweating and increased blood pressure. This scale assesses how the person is feeling at a specific time, the higher the score the greater feeling of apprehension, tension, nervousness and annoyance. Trait-anxiety refers to individual tendency to react to perceived situations as threatening with anxiety [36].

Anxiety symptoms according to STAI scores were defined as: low (<33), medium (33–49) and high (> 49) [16]. The Brazilian Portuguese version of this inventory demonstrates adequate reliability and validity [34, 37]. In the present study the STAI had a Cronbach’s alpha of 0,93 for trait-anxiety and 0,92 for the state-anxiety scales.

### Study variables

We analyzed sex, age, years of the medical training, school legal status (public or private), and school location (state capital or other cities), tuition scholarship, housing (alone or with someone), support service, BDI and STAI scores. In Brazil, the Medical degree is obtained in a 6 years undergraduate program and it is generally stratified into three periods: basic sciences (1^st^ and 2^nd^ years), clinical sciences (3^rd^ and 4^th^ years) and clerkship (5^th^ and 6^th^ years). We respected this classification in our study.

### Statistical analysis

Categorical variables are presented as proportions and continuous variables as mean ± standard deviation. Chi-squared and Kruskal-Wallis tests were used whenever applicable. We built multinomial logistic regression models to study whether age, sex, housing accommodations, year of medical training, school legal status (public or private), school location (state capital or other cities), and tuition scholarship were associated with depressive symptoms, state-anxiety or trait-anxiety. All models included age, sex and year of medical training as independent variables; so all results are adjusted for these characteristics. Assessing multicollinearity directly from multinomial models yields results of very difficult interpretation. Therefore, we assessed multicollinearity among the independent variables in all models calculating the variance inflation factors (VIF) of correspondent linear models. In these linear models, the independent variables were the same used in the multinomial models. In all cases, VIF values were below 1.4, showing there was no substantial multicollinearity among the independent variables. Statistical analysis was performed on R software version 3.1.1 (Vienna, Austria). Significance level was set at 0.05.

## Results

In this sample of 1,350 medical students (response rate 81.8 %) [30–32]. 557 (41.3 %) individuals had a BDI score of 10 points or higher, indicating the presence of mild depressive symptoms, at least. Additionally, 1,103 (81.7 %) and 1,155 (85.6 %) students had STAI scores above the threshold for moderate state and trait anxiety symptoms, respectively. Sample distributions of BDI and STAI scores according to socio-demographic variables are shown in Tables 1 and 2, respectively. In bivariate analyses, female students (*p* < 0.001) and students from schools located in capital cities (*p* = 0.001) referred more depressive symptoms. State anxiety symptoms were also more frequent in females (*p* < 0.001). Trait anxiety was more frequent in females (*p* < 0.001) and in students living in capital cities (*p* = 0.026). We did not find significant differences when years of the medical school were taken into account for depression (*p* = 0.859), state anxiety (*p* = 0.624) and trait anxiety (*p* = 0.4267) symptoms.

**Table 1:** Sociodemographic characteristics of study sample according to the levels of depressive symptoms

		Depression symptoms		
	Total	None	Mild	Moderate/Severe
Total	1350	793(58.7 %)	385(28.5 %)	172(12.8 %)
Age (years) mean ± SD)	22.8 (3.0)	22.8 (3.0)	22.8 (3.2)	22.7 (2.9)
Sex				
Female	714	365(51.1 %)	242(33.9 %)	107(15.0 %)
Male	636	428 (67.3 %)	143(22.5 %)	65(10.2 %)
Year of medical school				
1st/2nd (basic sciences)	459	269(58.6 %)	129(28.1 %)	61(13.3 %)
3rd/4th (clinical sciences)	491	283(57.6 %)	142(28.9 %)	66(13.4 %)
5th/6th (clerkship)	400	241(60.2 %)	114(28.5 %)	45(11.2 %)
School type				
Public School	882	516(58.5 %)	242(25.4 %)	124(14.1 %)
Private School	468	277(59.2 %)	143(30.6 %)	48(10.3 %)
School location				
Capital city	782	428(54.7 %)	239(30.6 %)	115(14.7 %)
Other cities	568	365(64.3 %)	146(25.7 %)	57(10.0 %)
Financial aid program				
None	1115	664(59.6 %)	312(28.0 %)	139(12.5 %)
Scholarship student	235	129(54.9 %)	73(31.1 %)	33(14.0 %)
Housing				
Alone	258	161(62.4 %)	61(23.6 %)	36(14.0 %)
With someone	1092	632(57.9 %)	324(29.7 %)	136(12.5 %)
- with parents	565	324(57.3 %)	166(29.4 %)	75(13.3 %)
- with other family members	135	80(59.3 %)	42(31.1 %)	13(9.6 %)
- with friend(s)	328	196(59.8 %)	97(29.6 %)	35(10.7 %)
- with partner	27	14(51.9 %)	8(29.6 %)	5(18.5 %)
- with spouse and children	11	5(45.5 %)	2(18.2 %)	4(36.4 %)
- others	26	13(50.0 %)	9(34.6 %)	4(15.4 %)

**Table 2:** Characteristics of study sample according to the levels of state and trait anxiety symptoms

	Anxiety-state			Anxiety-trait		
	None to low	Medium	High	None to low	Medium	High
Total	247 (18.3 %)	693(51.3 %)	410(30.4 %)	195(14.4 %)	675(50 %)	410(30.4 %)
Age (mean ± SD)	22.7 (3.3)	22.8 (2.8)	22.8 (3.2)	23.2 (3.5)	22.8 (2.8)	22.6 (3.1)
Sex						
Female	103 (14.4 %)	353 (49.4 %)	258 (36.1 %)	78(10.9 %)	330(46.2 %)	306(42.9 %)
Male	144 (22.6 %)	340 (53.5 %)	152 (23.9 %)	117(18.4 %)	345(54.2 %)	174(27.4 %)
Year of medical school						
1st/2nd	90(19.6 %)	223(48.6 %)	146(31.8 %)	64(13.9 %)	218(47.5 %)	177(38.6 %)
3rd/4th	88(17.9 %)	254(51.7 %)	149(30.3 %)	67(13.6 %)	257(52.3 %)	167(34.0 %)
5th/6th	69(17.2 %)	216(54.0 %)	115(28.7 %)	64(16.0 %)	200(50.0 %)	136(34.0 %)
School type						
Public School	162(18.4 %)	449(50.9 %)	271(30.7 %)	132(15.0 %)	421(47.7 %)	329(37.3 %)
Private School	85(18.2 %)	244(52.1 %)	139(29.7 %)	63(13.5 %)	254(54.3 %)	151(32.3 %)
School location						
Capital city	130(16.6 %)	398(50.9 %)	254(32.5 %)	96(12.3 %)	397(50.8 %)	289(37.0 %)
Other cities	117(20.6 %)	295(51.9 %)	156(27.5 %)	99(17.4 %)	278(48.9 %)	191(33.6 %)
Financial aid program						
None	217(19.5 %)	564(50.6 %)	334(30.0 %)	167(15.0 %)	553(49.6 %)	395(35.4 %)
Scholarship student	30(12.8 %)	129(54.9 %)	76(32.3 %)	28(11.9 %)	122(51.9 %)	85(36.2 %)
Housing						
Alone	43(16.7 %)	138(53.5 %)	77(29.8 %)	42(16.3 %)	128(49.6 %)	88(34.1 %)
with someone	204(18.7 %)	555(50.8 %)	333(30.5 %)	153(14.0 %)	547(50.1 %)	392(35.9 %)
with parents	100(17.7 %)	290(51.3 %)	175(31.0 %)	75(13.3 %)	289(51.2 %)	201(35.6 %)
with other family members	21(15.6 %)	77(57.0 %)	37(27.4 %)	13(9.6 %)	68(50.4 %)	54(40.0 %)
- with friend(s)	67(20.4 %)	162(49.4 %)	99(30.2 %)	54(16.5 %)	163(49.7 %)	111(33.8 %)
- with partner	8(29.6 %)	9(48.1 %)	6(22.2 %)	5(18.5 %)	14(51.9 %)	8(29.6 %)
- with spouse and children	2(18.2 %)	13(36.4 %)	5(45.5 %)	3(27.3 %)	2(18.2 %)	6(54.5 %)
- others	6(23.1 %)	9(35.6 %)	11(42.3 %)	3(11.5 %)	11(42.3 %)	12(46.2 %)

Table 3 describes the coexistence of depression and anxiety symptoms. Individuals with depression are more prone to present state and/or trait anxiety symptoms. High state anxiety scores are present in 14,4 %, 43,9 % and 73,8 % of participants with no, mild and moderate/severe depression, respectively. High trait anxiety scores are present in 15,3 %, 53,0 % and 90,1 % of participants with no, mild and moderate/severe depression respectively. A substantial number of participants have coexistence of those conditions. We found that 165 (12.2 %) individuals had simultaneously moderate to severe depressive symptoms and medium to high state anxiety symptoms, and 171 (12.7 %) individuals had moderate to severe depressive symptoms and medium to high trait anxiety symptoms.

**Table 3:** Coexistence of depression and anxiety symptoms according to BDI and STAI scores

		Depression symptoms			
		None	Mild	Moderate/Severe	*p*
Anxiety state	None to low	215 (27.1)	25 (1.9 %)	7 (0.5 %)	<0.001
	Medium	464 (34.4 %)	191 (14.1 %)	38 (2.8 %)	
	High	114 (8.4 %)	169 (12.5 %)	127 (9.4 %)	
Anxiety trait	None to low	190 (14.1 %)	4 (0.3 %)	1 (0.1 %)	<0.001
	Medium	482 (35.7 %)	177 (13.1 %)	16 (1.2 %)	
	High	121 (9 %)	204 (15.1 %)	155 (11.5 %)	

Table 4 shows the results of multinomial logistic regression models for the association between students or schools’ characteristics and depressive, state anxiety and/or trait anxiety scores. Female sex was associated with higher depressive, state anxiety and trait anxiety scores. We also found a significant, dose-effect direct association between studying in schools in capital cities and both depressive symptoms and trait anxiety scores. In addition, we also found a significant positive association between schools in capital cities and the highest level of state anxiety symptoms. Benefits from financial aid programs offering tuition was positively associated with state anxiety, but not with trait anxiety or depressive symptoms.

**Table 4:** Association among sociodemographic characteristics of study sample, depression, state- and trait- anxiety symptoms

		Depression symptoms		Anxiety-state		Anxiety- trait	
		Mild	Moderate/Severe	Medium	High	Medium	High
Model 1	Age (per 1 year increase)	1.02 (0,97-1.07)	1.01 (0.95–1.08)	0.99 (0.94–1.05)	1.02 (0.96–1.08)	0.95 (0.9–1.01)	0.95 (0.89–1.01)
	Sex (female)	2.00 (1.56–2.57)	1.94 (1.38–2.72)	1.45 (1.08–1.95)	2.39 (1.73–3.3)	1.41 (1.02–1.95)	2.58 (1.83–3.64)
	Sex (male)						
		Ref (1.0)	Ref (1.0)	Ref (1.0)	Ref (1.0)	Ref (1.0)	Ref (1.0)
	Year of medical school						
	3rd/4th	1.02 (0.75–1.39)	1.02 (0.68–1.53)	1.19 (0.83–1.7)	1.02 (0.69–1.52)	1.23 (0.82–1.83)	0.99 (0.65–1.51)
	5th/6th	0.93 (0.65–1.34)	0.8 (0.49–1.3)	1.31 (0.86–1.99)	0.99 (0.62–1.56)	1.1 (0.7–1.72)	0.94 (0.59–1.51)
	1st/2nd	Ref (1.0)	Ref (1.0)	Ref (1.0)	Ref (1.0)	Ref (1.0)	Ref (1.0)
Model 2	School type						
	Public school	0.92 (0.71–1.19)	1.42 (0.98–2.04)	0.96 (0.7–1.3)	1.03 (0.74–1.45)	0.79 (0.56–1.11)	1.06 (0.74–1.52)
	Private school						
		Ref (1.0)	Ref (1.0)	Ref (1.0)	Ref (1.0)	Ref (1.0)	Ref (1.0)
Model 3	School location						
	Capital city	1.39 (1.08–1.79)	1.73 (1.22–2.45)	1.21 (0.9–1.62)	1.45 (1.05–2.01)	1.49 (1.08–2.06)	1.57 (1.12–2.21)
	Other cities						
		Ref (1.0)	Ref (1.0)	Ref (1.0)	Ref (1.0)	Ref (1.0)	Ref (1.0)
Model 4	Financial aid program						
	Scholarship student	1.20 (0.87–1.66)	1.20 (0.78–1.85)	1.74 (1.13–2.69)	1.66 (1.04–2.65)	1.38 (0.88–2.18)	1.32 (0.82–2.13)
	None						
		Ref (1.0)	Ref (1.0)	Ref (1.0)	Ref (1.0)	Ref (1.0)	Ref (1.0)
Model 5	Housing						
	Alone with someone	0.74 (0.54–1.03)	1.06 (0.7–1.59)	1.18 (0.81–1.72)	1.11 (0.73–1.69)	0.86 (0.58–1.28)	0.83 (0.55–1.27)
		Ref (1.0)	Ref (1.0)	Ref (1.0)	Ref (1.0)	Ref (1.0)	Ref (1.0)

Only 342 (25.3 %) participants agreed to the statement “I have adequate access to psychological care” (statement 1) while 153 (11.3 %) participants agreed with the statement “There is a good support system for students who get stressed” (statement 2) (Table 5). It is noteworthy these concordance rates were even lower in individuals with more anxiety and depressive symptoms. We observed a significant trend for lower concordance with the statement 1 in individuals with more prominent state (p for trend < 0.001) and trait (p for trend = 0.019) anxiety symptoms. A similar, but non-significant trend (p for trend = 0.056) was also observed for the association with increasingly depressive symptoms. For the statement 2 there was a statistically significant trend for lower concordance rates in individuals with more intense depressive (p for trend = 0.033), state anxiety (p for trend = 0.003) and trait-anxiety (p for trend = 0.020) symptoms.

**Table 5:** Concordance rates with the sentences “I have adequate access to psychological care” and “There is a good support system for students who get stressed” according to the levels of depressive and anxiety symptoms

		“I have adequate access to psychological care”	“There is a good support system for students who get stressed”
Total		342 (25.3 %)	153 (11.3 %)
Depression symptoms	None	222 (28.0 %)	104 (13.1 %)
	Mild	77 (20.0 %)	33 (8.6 %)
	Moderate to severe	43 (25.0 %)	16 (9.3 %)
Anxiety trait	None to low	74 (37.9 %)	31 (15.9 %)
	Medium	147 (21.8 %)	77 (11.4 %)
	High	121 (25.2 %)	45 (9.4 %)
Anxiety state	None to low	86 (34.8 %)	38 (15.4 %)
	Medium	165 (23.8 %)	82 (11.8 %)
	High	91 (22.2 %)	33 (8.0 %)

## Discussion

We found a high prevalence of depressive and anxiety symptoms among Brazilian medical students. A substantial number of students had coexistent anxiety and depressive symptoms. Females, tuition scholarship students and students from medical schools located in capital cities were more prone to have anxiety and/or depressive symptoms. According to students’ perceptions, the access to psychological care and support is not sufficient.

The prevalence of depressive symptoms in Brazilian medical students (41.3 %) is higher than the global prevalence (28.0 %) recently estimated by a meta-analysis of 62 728 medical students and 1,845 non-medical students pooled across 77 studies (95 % confidence interval [CI] 24.2–32.1 %) [1]. Our findings of a high prevalence of state-anxiety (81.7 %) and trait-anxiety (85.6 %) in medical students are consistent with previous studies [21, 38, 39]. There are evidences that depression and mean trait-anxiety scores in medical students are even higher when compared to age-matched controls in the general population [20, 21]. However, the high trait-anxiety prevalence found in present study is similar to that reported in Brazilian age-matched undergraduate students [37, 40]. High depression prevalence was reported among students of humanities, exact sciences [41] and health services [42]. According to the literature it continuous unclear if depression and anxiety symptoms is more common in medical students than non-medical [22, 38].

We found a high coexistence of depressive and anxiety symptoms among medical students. In the 1980s some researchers questioned if anxiety and depression could be reliably differentiated using STAI and BDI [43, 44]. Currently there are consistent evidences of the adequate psychometrics properties of both BDI and STAI scales [34, 45, 46]. These results are consistent to the epidemiological studies that shown major depressive disorder has high comorbidity with numerous anxiety disorders in general population [20, 21].

Our data showed that female medical students were more prone to have depressive and anxiety symptoms than males. Comparisons of depressive and anxiety symptoms by gender among medical students yielded mixed findings showing either no difference or high prevalence among female medical students [1–3]. The higher prevalence of depression in female medical students has multiple explanations, including cultural aspects related to social stigma and gender inequity [39, 47], personality traits [7, 48], conflicting role demands [48], and medical educational environment [23–25, 47, 50]. An important factor to be considered is the medical education practices. Evidences shown that the educational environment has a significant impact on the well being of medical students [50]. A recently study showed that female medical students feel more discouraged and tired in medical training than the male colleagues and also reported greater solitude and a more negative perception of their social life [32]. The adaptation in medical schools that are no longer exclusively masculine with education practices that support a dominant patriarchy culture, seems to have a high psychological cost for women [47–51]. In Brazilian the proportion of females in medical schools increased in recent years from 46.3 % of 47 386 applicants in 1995 to 55.6 % in 2011 [52]. Although women are worldwide majority in medical schools and medical workforce there is inequity of opportunities in academic and across the professional [53].

In our study tuition scholarship students showed state-anxiety, but not trait-anxiety or depressive symptoms. Hojat et al. reported that among first- and second-year students at the Jefferson Medical College, 42 % had experienced financial problems in the previous 12 months and considered it as a stressful life event [48]. Wege et al. 2016 reported the association between financial hardships with poor mental health and psychosomatic symptoms [4].

Entering in medical undergraduation required to the students changing their lifestyles [25, 54]. One of these changes is living faraway from families and friends outside their hometowns. In this case housing accommodations (alone or with peers) can impact the students’ well-being and quality of life during the medical training [25]. Our hypothesis that students who live alone have higher depression and anxiety scores was not confirmed. Furthermore we confirmed the hypotheses that students from medical schools located in capital cities showed higher depression and anxiety scores. This suggests some factors related to the lifestyle more common in capital cities, like traffic, violence, may play a role in student mental health [55].

Related to institutional factors associated with anxiety and depression prevalence, we found no significant difference among years of the medical school, in contrast to previous studies [2, 25, 40]. Vitalino et al. reported that the number of depressed and anxiety students increased at the end of the first semester [40]. In the otherwise Ball and Bax noted BDI scores peaked in mid semester and returned to baseline by the end of the semester [54]. Longitudinal studies which compared the 4 years of American medical school reported that the depression scores peaked in the end of the second year but remained higher than baseline among fourth-year students [56, 57]. Differences in study populations may be responsible for these conflicting findings. On the other hand, those studies had convenience samples, and we could speculate that volunteer students may be those facing greater suffering along the medical training or have a more critical view when compared to randomly sampled students.

Another institutional factor was the access to psychological support, students with more depression and anxiety symptoms disagree more than their peers with the statements “I have adequate access to psychological support” and “There is a good program to stress in my school”. Hillis et al. reported that most of the students (71 %) knew about support services available in their schools, although few of them reported that services were properly offered [58]. This could suggest that medical students with more depression and anxiety symptoms either have less access to a psychological support or/and perceive it as adequate.

The strengths of this study are that it is consisted of a large, multicenter, randomly selected sample, from Brazilian schools located in all regions of the country and with a high response rate. We used validated questionnaires to address anxiety and depression symptoms. Our results must be interpreted in their context also. Our study has a cross-sectional design, which does not allow inferences of causality. Our sample was restricted to Brazilian medical students, and differences in study populations require caution to extend its findings to other settings.

Our findings offer evidences to drive interventions to deal with personal and institutional factors that affect medical students’ mental health, especially among females and students with financial hardships. These evidences suggest that medical schools should development programs to promote gender and social equity and strategies to improve psychological support services.

The comprehension of anxiety and depression in medical undergraduation context can be a step to improve educational environment, change habits and help the development of the new generation of physicians. There is growing literature on the health and well-being, yet few studies about medical students’ anxiety. A meta-analysis on anxiety among medical students would contribute with a global overview, along to longitudinal studies to establish causality.

## Conclusions

We found a high prevalence of anxiety and depressive symptoms in VERAS study participants. The factors associated with the increase of medical students’ depression and/or anxiety symptoms were female sex, school location and financial problems. Regarding to the years of the medical school we found no significant difference. According to students’ perceptions, the access to psychological care and support is not sufficient.

## Abbreviations

BDI: Beck Depression Inventory
STAI: State-Trait Anxiety Inventory
VERAS: Vida do estudante e residente da área da saúde (Life os Students and Residents from Health Professions)
